# Electric Field and Current Transport Mechanisms in Schottky CdTe X-ray Detectors under Perturbing Optical Radiation

**DOI:** 10.3390/s130709414

**Published:** 2013-07-22

**Authors:** Adriano Cola, Isabella Farella

**Affiliations:** Institute for Microelectronics and Microsystems (CNR-IMM), Via Monteroni, Lecce 73100, Italy; E-Mail: isabella.farella@le.imm.cnr.it

**Keywords:** CdTe, X-ray detectors, Pockels effect, deep levels, carrier transport, polarization, Schottky barrier, trap filling, carrier injection

## Abstract

Schottky CdTe X-ray detectors exhibit excellent spectroscopic performance but suffer from instabilities. Hence it is of extreme relevance to investigate their electrical properties. A systematic study of the electric field distribution and the current flowing in such detectors under optical perturbations is presented here. The detector response is explored by varying experimental parameters, such as voltage, temperature, and radiation wavelength. The strongest perturbation is observed under 850 nm irradiation, bulk carrier recombination becoming effective there. Cathode and anode irradiations evidence the crucial role of the contacts, the cathode being Ohmic and the anode blocking. In particular, under irradiation of the cathode, charge injection occurs and peculiar kinks, typical of trap filling, are observed both in the current-voltage characteristic and during transients. The simultaneous access to the electric field and the current highlights the correlation between free and fixed charges, and unveils carrier transport/collection mechanisms otherwise hidden.

## 1. Introduction

Understanding charge carrier transport in radiation detectors is of extreme relevance as detectors rely on the efficient collection of photo-generated charge pairs. High electric fields over several mms thickness with low leakage currents are basic requirements for efficiently detecting X- and -photons, and this involves the use of semiinsulating (SI) materials and suitable contacts.

In this regard, Schottky detectors based on high quality SI CdTe have been produced for more than ten years; they sustain high operating voltages with sufficiently low currents and show excellent spectroscopic performance [1]. However transient phenomena known as *bias induced polarization* seriously degrade their operation: under bias, a space charge builds up which eventually shrinks the electric field under the blocking contact [2,3]. Additionally, the current exhibits non-monotonic transients, as recently shown by Principato *et al.* [4].

Under high X-ray radiation flux, all CdTe detectors, both with Ohmic and Schottky contacts, also experience a reduction in counting rate and spectroscopic performance with time [5–7]. This *radiation induced polarization*, still related to a space charge build-up, and therefore to a strong distortion of the electric field distribution, is additionally characterized by a progressive decrease of the carrier lifetime [7].

Optical excitation provides an effective way to investigate the mechanisms of polarization and, more generally, to investigate the carrier transport mechanisms within the detectors under perturbing conditions [8–10]. Major advantages consist in the possibility to accurately control the photon penetration depth and incident intensity. Also, carrier generation rates comparable to those produced by X-ray sources can be obtained along the detector. Furthermore, when sub-bandgap radiation is used, deep level occupation can be directly altered, with consequence on the electric field distribution [10]. Eventually, the perturbation by optical radiation of suitable wavelength can be conceived to counterbalance the detrimental effect of high X-ray fluxes [9,11,12].

When considering the detector electrical properties, key players are fixed charges, traps and contacts and, under irradiation, the photo-generated carriers. Under dark conditions, the number of free carriers is pretty low, but under irradiation free carriers in part of the detector can be comparable or greater than fixed charges. All such ingredients are related by the basic transport equations, *i.e.*, the Poisson and current equations which, under the proper boundary conditions provided by the contacts, determine the fundamental physical quantities which can be experimentally accessed, *i.e.*, the electric field and the flowing current. These are also the quantities investigated in the present work.

A powerful technique able to probe the electric-field distribution, with good spatial and temporal resolution, is based on the so-called Pockels effect. It has shown to be very effective when applied to CdTe/CdZnTe detectors [13–19], due to the large electro-optic coefficient of those materials and the limited perturbation of the field itself.

Recently, the Time of Flight (ToF) technique has been also used to extract electric field profiles [20,21]. However, ToF merges the information of the electric field with the charge carrier transport along it, making the electric field calculation less direct, especially in presence of trapping.

All the exposed considerations motivate the scope of this work and the chosen approach. We use optical radiation of different wavelengths (above and below the band gap) to identify the mechanisms perturbing the electrical properties of Schottky CdTe detectors, by measuring the electric field distributions by Pockels effect and the photocurrent, under various experimental conditions.

The work is organized as follows: Section 2 describes the experiments, in particular the Pockels effect experimental set-up for the electric field evaluation. General considerations concerning optical irradiation of CdTe detectors are reported in Section 3. The effects of perturbation are analysed in Sections 4 and 5, which refer to irradiations through anode and cathode, respectively. In both cases, results are gathered for wavelengths in the range from 700 to 820 nm (highly absorbed radiation), and also for wavelengths in the range 900–950 nm (weakly absorbed radiation). Larger and qualitatively different perturbation is reported for the 850 nm wavelength, which approximately corresponds to the CdTe band edge. Correlations between the photocurrent and the electric field distribution are presented both for measurements as a function of voltage, and temperature and for transients. The role of contacts is extensively discussed throughout the paper and, in particular, in Section 6 where the relation between injection mechanisms and photocurrent is tackled.

## 2. Experimental

**Figure 1.**
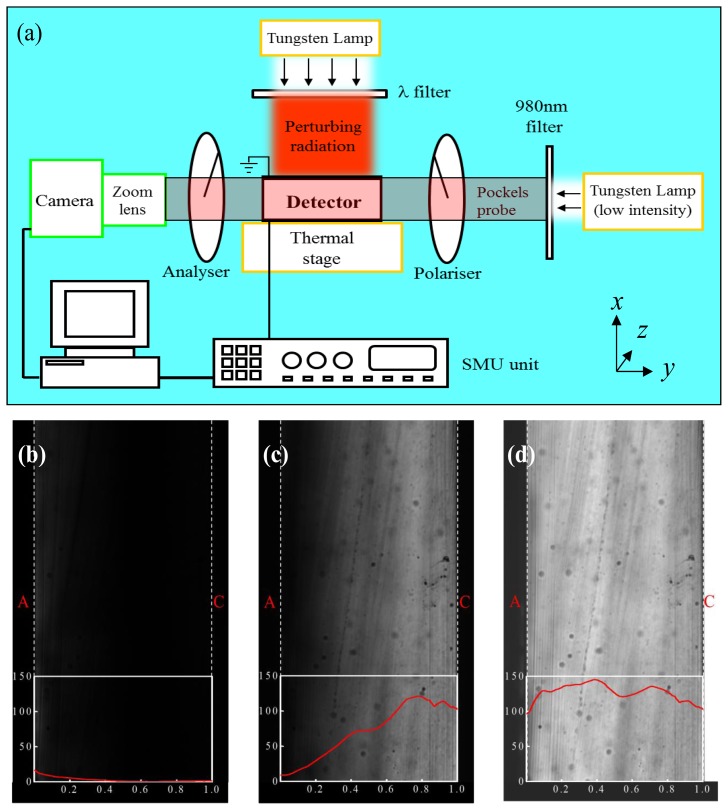
(**a**) Experimental set-up for Pockels effect measurements under optical perturbation. Typical Pockels image (polarisers crossed) of the unbiased detector (**b**) and (**c**) biased at 600 V in perturbation-free condition at T = 25 °C. Superimposed the intensity profile *I* (*y*) from anode (A) to cathode (C), calculated by averaging *I* (*x*, *y*) along the *x*-direction. (**d**) Image of the unbiased detector, with parallel polarisers (*I_0_* (*x*, *y*)). Superimposed the intensity profile *I_0_* (*y*) from anode (A) to cathode (C), calculated by averaging *I_0_* (*x*, *y*) along the *x*-direction. The corresponding electric field profile *E* (*y*) is shown in Figure 3 (dark squares). The dark spots visible in the images can be ascribed to Te inclusions/precipitates in CdTe crystal, and streaks to dicing crystal procedure.

**Figure 2.**
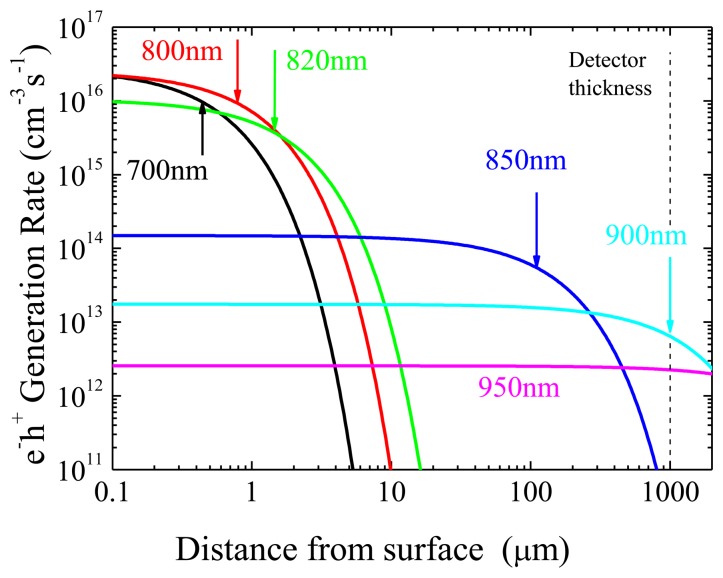
Spatial distribution of the electron-hole generation rate for different wavelengths, calculated assuming no reflections or contact absorption. The arrows denote the penetration depth for each wavelength. At 950 nm the penetration depth is about 2 mm. The dashed vertical line indicates the detector thickness (1 mm).

Pockels experiments have been performed using the crossed polariser technique in transversal configuration, where linearly polarised near infrared radiation (NIR) impinges perpendicular to the lateral surface of the detector, thus parallel to the contacts. By passing through the detector, the radiation experiences the birefringence induced in the crystal by the applied voltage, and is detected after crossing a second polariser whose axis is perpendicular to the first one. The transmitted probe intensity is digitized by a charge coupled device with enhanced sensitivity in the NIR range, equipped with a 6× zoom lens and stored as an image. The experimental set-up is sketched in Figure 1a and typical Pockels images of the detector unbiased and biased (600 V) are shown in Figure 1b,c, respectively. For each (*x*, *y*) projection point of the detector side, the intensity *I* (*x*, *y*) of the transmitted light depends on the electric field distribution *E*(*x*, *y*) averaged along the optical path (*z*-direction), through the relation [22]:

(1) $$I(x,y)=I_{0}(x,y)\sin^{2}\left[\frac{\pi }{2}\frac{E(x,y)}{E_{0}}\right]$$

where *I*_0_ *(x, y)* is the transmitted intensity with polarisers parallel one to the other (see Figure 1d). The constant *E_0_* depends on material properties and size, and on the wavelength of the probe radiation, which is 980 nm in our case. The electric field profile *E (y)* from anode to cathode is calculated through Equation (1) by averaging *I* (*x*, *y*) and *I_0_* (*x*, *y*) along the *x*-direction, parallel to the electrodes (see Figure 2). Measurements have been carried out at different temperatures from 25 °C to 60 °C (stability ±0.1 °C), by placing the samples on a temperature controlled Instec HCP302 thermal plate. Polariser and analyser are oriented at 45° and -45°, respectively with respect to the (111) crystal orientation along which the external electric field is applied.

The NIR intensity has been kept as low as possible, and it was verified that the probe radiation did not affect at all the *E*-field distribution and, only weakly, the measured current. More details on the experimental set-up have been previously published [19]. As perturbing optical source, a 100 Watt tungsten lamp emitting a continuum spectrum has been used. We select the wavelength among a set of narrowband (10 nm FWHM) filters enabling transmission at different wavelengths, ranging from 700 to 950 nm. The beam has been expanded in order to uniformly illuminate the whole detector contact. Both irradiations through cathode and anode have been carried out. The optical power incident on the contacts at each wavelength has been measured by a calibrated photodiode. Values range from 2.6 mW to 3.9 mW, depending on the wavelength (from 700 to 950 nm). Note that the contact transparency is not known, and this hides the actual carrier generation rate into the detector.

Both voltage sweeps and transient experiments at fixed voltage have been performed using a Keithley 237 source-monitor unit while simultaneously recording Pockels images. In the voltage sweeps (up to 900 V), data acquisition occurs 1 second after each staircase voltage step (10 V) is applied. Transient measurements start at the end of an upward voltage staircase and they are performed at 600 V with 1 s sampling time. As for voltage sweeps and transient image acquisition, the optical perturbation was switched on few seconds before starting measurement with the detector unbiased. Measurements carried out during the voltage sweeps are not stationary, but approximately correspond to the first sampled data acquired during transients. Measurements refer to an Acrorad planar detector 10 × 10 mm^2^ in size, 1 mm thick, but similar results have been found on other nominally equal Acrorad detectors. The material is a high quality (111)-oriented Cl doped SI (resistivity ∼10^9^**Ω** cm), slightly *p*-type CdTe single crystal, grown by Traveling Heater Method. The hole-blocking anode contact is formed by depositing In on the Te-face of the crystal, followed by a Ti layer; the Pt contact, electroless deposited on the Cd-rich opposite face, acts as an Ohmic cathode.

## 3. Optical Irradiation: General Considerations

Measured absorption coefficients [23] and incident optical power make it possible to roughly estimate the spatial distribution of the carrier generation rate *G*, at each considered wavelength (see Figure 2). For *λ* ≤ 820 nm, a huge concentration of carriers is generated in a width thinner than 2 μm. As previous ToF measurements have pointed out [23], this thin region is quite defected, and pledged to recombination processes. A much more penetrating radiation is that at *λ* = 850 nm, (CdTe absorption coefficient is very steep around this wavelength) but it is still absorbed in about 1/10 (110 μm) of the detector thickness. In terms of the statistical distribution of G, we evaluate that the 850 nm irradiation is equivalent to a flux of 1.7 × 10^10^ photons /(mm^2^s) at 42 keV energy. This comparison is based, as the spatial distribution is concerned, on similar absorption coefficients of 850 nm and 42 keV photons. With regard to the X-ray flux, the value is calculated assuming an equal number of photo-generated carriers, *i.e.*, one electron-hole pair each photon at 850 nm and 4.43 eV [24] to create a pair for the X-rays. At longer wavelengths, the penetration depth [25] becomes comparable with the detectors thickness (*λ* = 900 nm) or even greater (*λ* = 950 nm); hence, at such wavelengths, *G* is relatively low and uniform across the detector.

The strong absorption for *λ* < 820 nm implies, from Ramo's theorem [26], that only those carriers moving away from the irradiated contact will contribute to the charge transport, the others being quickly collected from the same contact. However, a previous study [23] showed that only a fraction of the photo-generated carriers can escape the thin defected region involved in the photo-generation.

At 850 nm, due to the greater penetration depth, a large number of carriers of both types will contribute to the transport without being strongly affected by the surface defects. Finally, for longer wavelengths, the electron-hole direct generation probability decreases and the low absorption is due to bulk defects. In this case, each optical transition does generate only one kind of free carriers, depending on the optical capture cross section, on concentration of defects and their carrier occupancy statistics.

Clearly, we are overestimating the actual carrier generation inside the detector because of the reflection from the surface and, overall, because of the optical attenuation from metal contacts, which is unknown. Hence, the rate *G* reported in Figure 2 has the only aim to highlight the relative variations of the carrier generation rate and the range of penetration depths at different wavelengths.

In the following, we present results for irradiation on the anode and on the cathode surface. Particularly at short wavelengths, we expect different perturbations, because of the different carrier transport properties of electrons and holes, and because of the different nature of the two contacts (Ohmic and Schottky), resulting also in a non-uniform distribution of the electric field.

## 4. Anode Irradiation

### 4.1. Voltage Sweep Measurements

**Figure 3.**
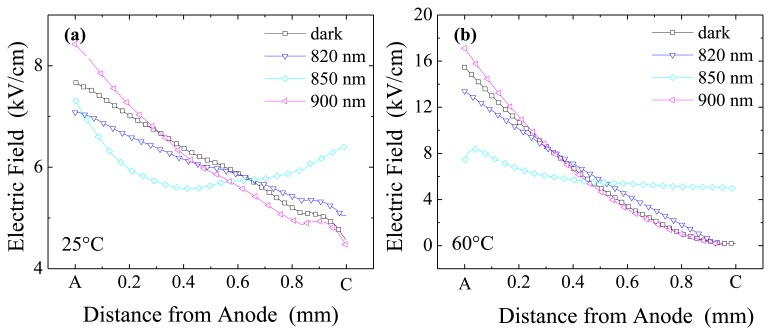
Electric field profiles at T = 25 °C (**a**) and T = 60 °C (**b**) under optical irradiation on the anode side: 820 nm, 850 nm, 900 nm. For comparison, the profile corresponding to no optical irradiation (dark) is also reported. Bias is 600 V.

We start reporting results obtained when irradiating anode side, *i.e.*, the hole blocking contact. In this configuration, at short wavelengths, the anode collects the nearby photo-generated electrons while the perturbing action is consigned to the photo-generated holes. Electric field profiles in Figure 3 refer to various wavelength irradiations, for two limit temperatures, 25 °C (Figure 3a) and 60 °C (Figure 3b). The applied voltage is 600 V, the Pockels data being acquired during voltage sweeps.

At 25 °C, the effect of optical perturbation is evident at 850 nm, where the field becomes slightly concave. On the contrary, at the other wavelengths, it is still linear but slightly changes its slope with respect to the dark case. In particular, at *λ* = 820 nm, and less at 700 nm (not shown), anode irradiation causes a little reduction in field slope while at *λ* = 900 nm, and less at 950 nm (not shown), an increase is observed. The field slope decrease observed at short wavelength has to be associated with a decrease of the (negative) space charge caused by the capture of the free holes that drift from the irradiated anode to the cathode.

It has been previously shown [27] that, without optical perturbation, a temperature increase results in a field slope increase. Such a behaviour is also noticed under irradiation, except for *λ* = 850 nm: at this wavelength, the field is relatively insensitive to the temperature and changes occur only near the cathode. In Figure 3b, where the field profiles at 60 °C are reported, the trend previously observed at 25 °C is still maintained at the remaining wavelengths: for *λ* = 820 nm and *λ* = 900 nm the field slope is still lower and greater than perturbation-free case, respectively.

Hence, results at different temperatures confirm that, except at *λ* = 850 nm, the optical perturbation is not able to deeply affect the field distribution with respect to the non-irradiated case: the temperature increase of the field slope (*i.e.*, of the space charge) dominates due to the deep level emission. On the other hand, under 850 nm, the field becomes more uniform and relatively independent of the temperature.

**Figure 4.**
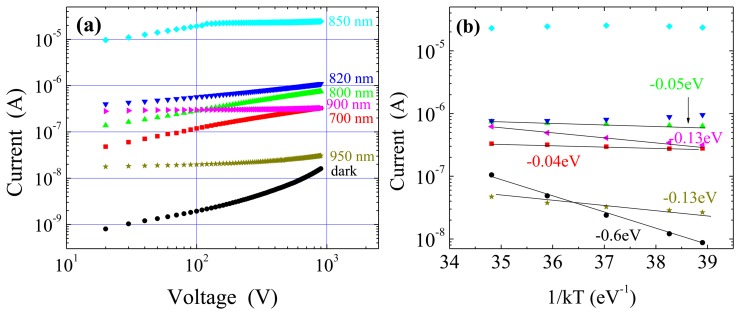
Optical irradiation on anode side: (**a**) photocurrent *vs.* voltage characteristics at different wavelengths, and under dark, T = 25 °C. (**b**) Thermal activation plot of photocurrent (and dark) at 600 V. Activation energies are also reported for each wavelength (same symbols and colours as (**a**)). At 820 and 850 nm a negligible activation energy is observed.

Photocurrent-voltage characteristics at 25 °C, under different perturbation wavelengths, are reported in Figure 4a. For all the curves, the dark current has been subtracted from the total measured current. From Figure 4a it can be seen that the photocurrent increases with the wavelength up to 850 nm, where the greatest values are measured. The photocurrent correlates well with the electric field perturbation: both of them increase with the wavelength since, at wavelengths more and more penetrating, a greater number of photo-generated carriers becomes effective. At 700 and 800 nm, we observe a photocurrent increase with the square root of the voltage; then the slope decreases at 820 nm and it nulls at 850 nm, where saturation occurs already for V > 100 V.

The results at 700 and 800 nm fit very well the prediction of a square root dependence of photocurrent on the voltage made by Zanichelli [28], who extended the Ramo's theorem to the case of a non-uniform electric field for photo-generation at the contact from which the field linearly decreases. Noticeably, Zanichelli's calculations implicitly assume no carrier injection from the illuminated contact and a negligible perturbation of the electric field, which are assumptions fulfilled in the present configuration.

From the Poisson equation, the distribution of the electric field depends on the total charge inside the detector. If non-perturbed, the fixed negative charge (∼10^11^ cm^-3^) from deep levels prevails over the free carriers. On the contrary, under continuous optical irradiation, photo-generated free carriers can affect the fixed charge through trapping or, if their concentration is high, they become prevalent with respect to the fixed charge. These two types of perturbation correspond to the irradiations in the 700 – 820 nm range, and at 850 nm, respectively.

It is well known that holes experience a strong trapping and therefore, at wavelengths in the 700-820 nm range, the small but appreciable decrease in the space charge should be related to the trapping of holes, which are moving from the irradiated anode to the collecting cathode. For such wavelengths, the space charge from the negatively charged deep levels still controls the field distribution, as the thermal behaviour of the field is basically like the unperturbed one.

In terms of current, however, the photo-generated holes greatly exceed the free carriers present in the non-perturbed case. Hence, the measured current is determined by the photo-generated holes subjected to trapping. Meanwhile the trapped holes contribute to decrease the negative space charge thus reducing the field slope. An enhancement of such effects is achieved by rising the wavelengths from 700 to 820 nm: because of the increase in the radiation penetration depth and the consequent reduction of the surface recombination, more photo-generated holes are allowed to escape from the surface region and can drift towards the cathode.

The situation changes at *λ* = 850 nm since a larger number of both electrons and holes are created well inside the detector (see Figure 2), and they do not suffer from the strong surface recombination. The presence of a concavity in the field distribution (Figure 3a) can be attributed to the presence of a net negative and positive charge, distributed on the anode and cathode side, respectively. The high photocurrent, and the strongly perturbed electric field, both independent of the temperature, confirm that free carriers directly affect the field distribution while the current is no longer limited by trapping, but rather by direct recombination. Hence, the large number of photo-generated electrons and holes prevails over the unperturbed space charge, and the field becomes controlled by the temperature independent recombination dynamics of the free carries. The observed saturation of the photocurrent will be discussed in more detail in Section 6.

A different mechanism occurs for longer wavelengths (900 nm and 950 nm). In such case, radiation absorption through the material is pretty low and involves transitions from/to deep levels. Hence, a small perturbation of the electric field, if appreciable, is expected as a consequence of a direct space charge modification. Previous experiments have shown that, for such wavelengths, both free electrons and holes are created throughout the detector and participate to the transport [23]. Present results indicate an overall increase of the negative space charge with respect to the perturbation-free case, as if the dominant mechanism was the optical excitation of an electron from the valence band to a deep level. Consistently with the low absorption, the measured photocurrent is quite low, and clearly saturates with the voltage, as already observed at 850 nm. However, a different recombination mechanism seems to dominate at 900 and 950 nm because of the limited number of free photo-generated carriers and their thermal behaviour. Indeed, the photocurrent values at 600 V as a function of 1/*kT*, see Figure 4b, show appreciable thermal variations only for *λ* = 900 nm and *λ* = 950 nm, with an activation energy of *E_a_* = -0.13 eV. These results suggest the involvement of deep levels not only in the field perturbation but also in the transport mechanism, since the photocurrent is due to recombination on the deep levels.

### 4.2. Transient Measurements

**Figure 5.**
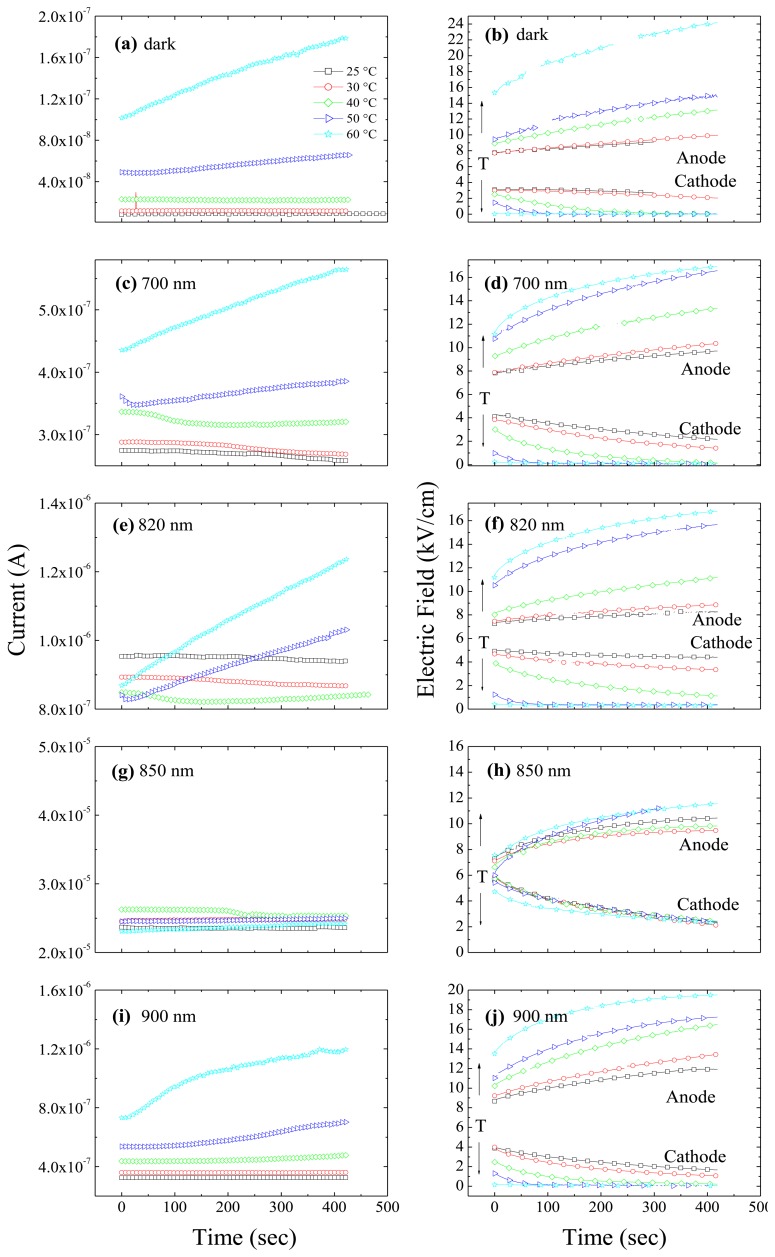
Transients of currents (Left) and electric fields at anode and cathode (Right) at different temperatures in perturbation-free condition (**a,b**), and under different optical irradiations on the anode side: 700 nm (**c**,**d**); 820 nm (**e**,**f**); 850nm (**g**,**h**); 900 nm (**i**,**j**). Applied voltage is 600 V.

In Figure 5 we report current transients and corresponding transients of the electric field at the anode and the cathode, for different temperatures and wavelengths. In a recent paper [27] we have shown that under dark conditions a strong correlation comes out between the current and the values of the electric field at the electrodes. Whenever the field near the Ohmic contact is not negligible, electrons will be injected, and drifted away by the field, thus becoming the main contribution to the flowing current. The transient regime originates from the deep level carrier emission which causes the electric field to become steeper. This corresponds to the decrease of the current and of the electric field at the cathode observed in Figure 5a,b for temperatures lower than 40 °C. For higher temperature, the transient increase of the current has to be connected to the considerable field enhancement at the anode. This makes predominant the hole contribution through the field dependent barrier height, as explained in Reference [27]. Under illumination, due to the large number of photo-generated carriers, photocurrent is much higher than dark current. Nonetheless, it is observed that a correlation still exists between the photocurrent transients and the field transients near the electrodes. At 700 nm (Figure 5c,d), it can be inferred that the current transients, even if quite limited, are related to the field at the cathode: at 25 °C and 30 °C both the field at the cathode and the current decrease. At higher temperature, 40 °C, after about 300 s, the field at the cathode becomes negligible: the current consistently shows a decrease in the first 200 s; then, as soon as the field at the cathode becomes pretty low, the current starts to increase (as a consequence of a field increase at the anode). At 50 °C we observe a similar behaviour, with the field at the cathode becoming negligible at about 50 s and the current showing a minimum few seconds before. At 60 °C the field at the cathode is already negligible at the starting time and the current thus increases, like the field at the anode. Similar phenomenology has been observed for the irradiation at 800 nm (not shown) and 820 nm (see Figure 5e,f). It is remarkable that electron injection at the cathode still affects the flowing current even when current is much larger than in the dark, and mainly due to the photo-generated holes drifting from the irradiated anode.

Under 850 nm, the photocurrent does not show any appreciable transient (see Figure 5g) or thermal activation (see Figure 4b). On the other hand, the electric field still shows increasing and decreasing transients at anode and cathode, respectively, which are not thermally activated (see Figure 5h). Even if the field tends to decrease at the cathode, it does not become negligible within the observation time (420 s). Hence, irradiation at 850 nm gives rise to a substantial different electric field behaviour with respect to the other wavelengths and with respect to the perturbation-free case: the field shrinks much less towards the anode and the deep level ionization does not become predominant at high temperatures. Hence, the electric field and the photocurrent are consistently independent of the temperature. It is presently unclear which mechanism produces transient effects of the electric field on a long time scale of hundreds of seconds, while the photocurrent keeps constant.

Only a limited increasing behaviour at high temperatures is observed in current transients under anode irradiation at *λ* = 900 nm (and 950 nm, not shown). Around room temperature, although the non-negligible field at the cathode is decreasing, the current remains constant.

## 5. Cathode Irradiation

### 5.1. Voltage Sweep Measurements

Both in terms of electric field and photocurrent, the effect of irradiating the cathode is in general enhanced and qualitatively different with respect to what happens on the anode side. Exception occurs for 900 and 950 nm irradiation where, as a consequence of the relatively uniform radiation absorption in the bulk, the effect of perturbation is similar for cathode and anode irradiations.

The high absorption coefficient and the presence of the Ohmic contact (Pt contact), which provides electron injection, can explain the behaviour observed for cathode irradiation at 700, 800 and 820 nm. It has been observed, in the case of anode irradiation, that the photo-generated holes are trapped and therefore contribute to decrease the negative space charge; in the present case, the photo-generated electrons, if trapped, contribute to increase the negative space charge and, therefore, the electric field slope. The increase of the field slope is indeed what is observed. However, electrons are not heavily affected by trapping, and the observed null field at the cathode can be rather attributed to the high concentration of photo-generated free electrons together with the Ohmic nature of that electrode. In fact, according to continuity equation, it is the large concentration of electrons near the cathode which forces the field to be negligible there.

Similarly to what observed for anode irradiation, at 850 nm, the field becomes concave, but now its value is much higher near the anode. At such wavelength, the different field distribution for cathode and anode irradiations is due to both the major role of electrons and holes (due to the limited radiation penetration depth) and from the non-uniform distribution of the unperturbed electric field.

**Figure 6.**
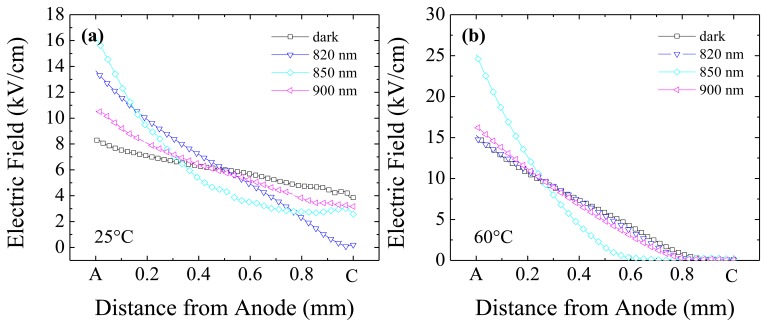
Electric field profiles at T = 25 °C (**a**), and T = 60 °C (**b**) under different optical irradiation conditions on the cathode side: 820 nm, 850 nm, 900 nm. For comparison, the profile corresponding to no optical irradiation is also reported. Bias is 600 V.

At 60 °C (Figure 6b) and all the perturbation wavelengths except 850 nm, the field distribution is quite similar to the unperturbed case: the field reduces almost linearly from quite high values at the anode to negligible ones at about 200 μm from the cathode. This finding indicates that, even more than for anode irradiation, at high temperature the optical perturbation effect becomes negligible, and carrier thermal emission from deep levels dominates.

Contrary to the relatively flat distribution observed for the anode irradiation, at 850 nm and 60 °C, now the field is strongly confined in half of the detector on the anode side. This behaviour is probably related to the large electron concentration which extends well inside the detector, from the irradiated cathode. However, as shown hereafter, the role of temperature on carrier injection is quite complex.

**Figure 7.**
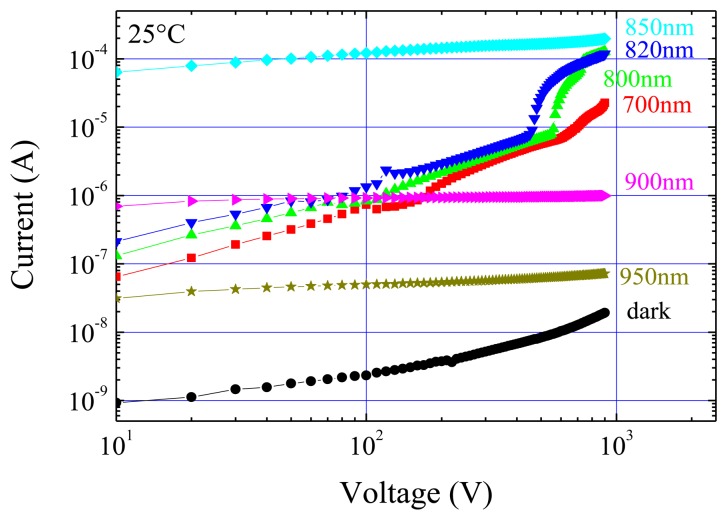
Optical irradiation on the cathode side: photocurrent *vs.* voltage at different wavelengths, and under dark. T = 25 °C.

In Figure 7 the room temperature photocurrents as a function of the applied voltage are reported for different wavelengths. Likewise anode irradiation case, dark current values have been subtracted from all the other curves. As expected, at 900 and 950 nm photocurrents are comparable with those corresponding to the anode case. At the other wavelengths, several important differences are evident with respect to the anode irradiation case. At the lowest wavelengths (700, 800, and 820 nm), depending on the wavelength, a kink is evident in the curves between 400 V and 600 V.

It has to be remarked that these features are similar to the space charge limited ones in insulating (or semiinsulating) materials when carrier injection is sufficient to fill the traps [29]. In our detector, in the perturbation-free case, such a condition is not achieved up to 900 V, even though some indications of carrier injection are provided by the quadratic behaviour of the current-voltage characteristics [27]. The optical carrier generation then facilitates the trap filling, allowing (together with the injecting nature of the contact) a lowering of the trap filling voltage. The same nature of the cathode also explains the large photocurrent values recorded at high voltages, being more than two orders of magnitude greater than that measured for the anode irradiation. Furthermore, as evident in Figure 7, the higher the wavelength the lower the voltage for the onset of the electron injection.

For *λ* = 850 nm the field is strongly perturbed and shows, near the cathode, appreciable values already at very low voltages. This accounts for the large photocurrent values recorded without the evidence of a kink even at low voltages.

**Figure 8.**
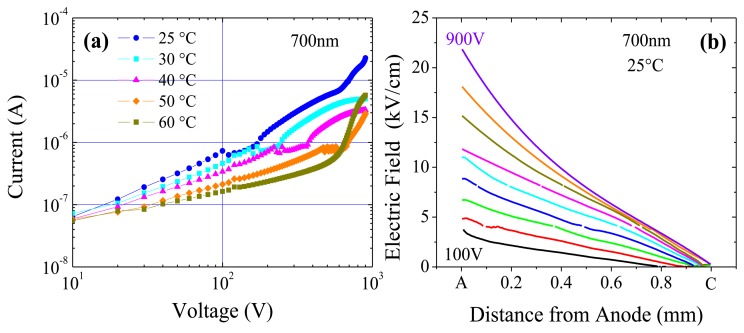
Optical irradiation at 700 nm on the cathode side: (**a**) Photocurrent at different temperatures. (**b**) Electric field profiles at different voltages, at 25 °C.

The effects of irradiation under 700, 800 and 820 nm present some common features which will be now investigated in detail. Measurements as a function of voltage (see Figure 8) indicate that current injection becomes efficient at applied voltages such that the field extends near to the cathode.

At low voltages, the photocurrent strongly depends on the field extension towards the cathode side: Figure 8a, where the photocurrent *vs.* voltage at different temperatures and λ = 700 nm is reported, shows that photocurrent lowers as temperature increases, according to a greater field slope. Moreover, as the applied voltage increases, a noisy almost saturated region appears, corresponding to current values of about 10^-6^ A; successively, it becomes evident the kink in the curve, which shifts to higher voltages as the temperature increases.

At 25 °C, it is observed that as the voltage increases, the profiles of the electric field try to extend more and more in the detector towards the cathode see (Figure 8b). However, the field remains negligible even at the highest voltages: as a consequence, an increase of the field slope is observed with the voltage. At 200 V, where the kink in the photocurrent appears, the field extends up to about 150 μm before the cathode. At higher temperatures, for example 50 °C, the kink in the photocurrent appears around 600 V which still corresponds to an extension of the field up to about 150 μm before the cathode. Therefore, it is clear that the field near the cathode triggers the injection of carriers.

The presence of the noisy and saturated region before the kink in the photocurrent curves, clearly observed at all temperatures except 60 °C, can be probably ascribed to transient effects that tend to shrink the field distribution against the opposite effect of the raising voltage. Important transients are indeed evident and are discussed in the next sub-section.

### 5.2. Transient Measurements

Similarly to what already observed in the anode irradiation case at the perturbing wavelengths of 700, 800 and 820 nm, for cathode irradiation, current transients exhibit peculiar features which can be correlated to the electric field transients.

**Figure 9.**
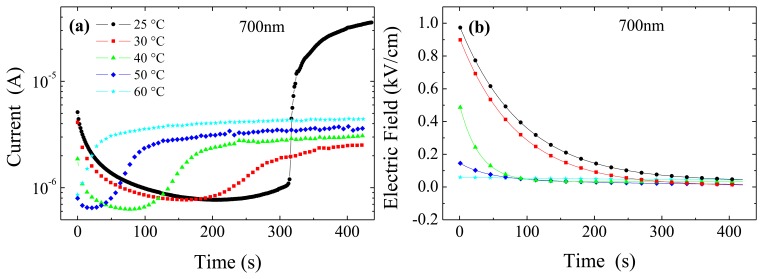
Transients at 600V, at different temperatures, for optical irradiation at 700 nm on the cathode side: (**a**) Current transients; (**b**) Electric field transients at 60 μm from the cathode.

In Figure 9a, current transients collected under cathode irradiation at 700 nm are reported. For different temperatures, some common characteristics can be noted: at the starting time, the current tends first to decrease till it becomes almost constant; then it increases and finally it almost saturates to a value which depends on the temperature. The decreasing current transient becomes shorter with temperature and eventually disappears for 60 °C. At 25 °C, after about 320 s, current experiences a sudden increase after which it tends to saturate.

As previously seen, the field at the cathode is negligible and therefore no apparent correlation exists with the photocurrent; however, the field distribution is changing with time, by recoiling from the cathode side and hence increasing at the anode side. A careful data inspection reveals that the initial decrease of the current closely resembles the decrease of the electric field at a specific distance from the cathode. In particular, Figure 9b, shows that both the time constant and the amplitude of the electric field at 60 μm from the cathode are remarkably correlated to the initial transient of the current.

**Figure 10.**
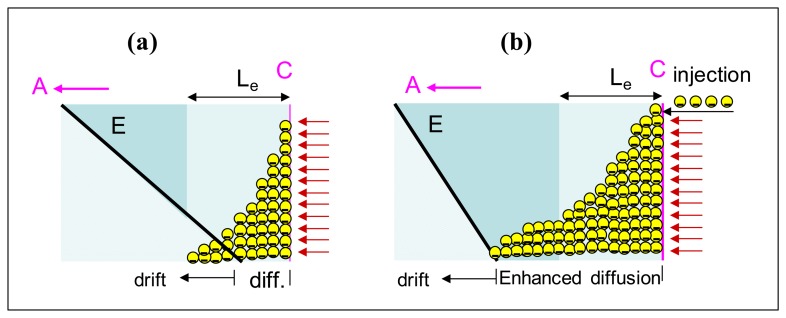
Sketch of transient effects near the irradiated cathode (highly absorbed radiation). In (**a**) the electric field is still close to the cathode and it is recoiling towards the anode (the current tends to decrease). In (**b**) the field has recoiled more than the L_e_ distance from the cathode and electron injection from cathode occurs causing enhanced diffusion (the current tends to increase and then to saturate).

To understand these results, it should be taken into account that the low field region close to the cathode enlarges with time; on the other hand, due to cathode irradiation, a large concentration of electrons are photo-generated very close to the cathode (holes are promptly collected). These electrons diffuse over their typical diffusion length, L_e_ = (D_e_τ_e_)^1/2^. Assuming an electron mobility of 1,100 cm^2^·V ^-1^·s ^-1^, from the measured μ_e_τ_e_ product (μ_e_τ_e_ = 1.3 × 10-^3^ cm^2^V^-1^) [23], we evaluate the electron trapping time τ_e_ = 1.2 × 10^-6^ s and the diffusion coefficient (from the Einstein relation). In the range 25-60 °C, L_e_ turns out to be 58-61 μm, hence photo-generated (and contact injected) electrons diffuse up to around 60 μm from cathode. During the transient, the electric field moves further away from cathode and the initial current decrease seems to correlate with the progressive reduction of the fraction of electrons drifted away by the electric field, as depicted in Figure 10a.

The subsequent increase observed in current transients is still related to electron injection and trap filling: when the low field region becomes comparable or even greater than L_e_, photo-generated electrons are hardly extracted; this can favour the filling of trap centres and then the increase of τ_e_. To some an extent, this mechanism should increase the effective diffusion length inside the region close to the cathode (Figure 10b).

We estimate that, when the current transient starts to increase, the extension of the low field region has reached a thickness of about 150 μm, a depth that we have already found to be critical for carrier injection. At this point the injecting contact supplies an additional amount of electrons. According to the enhanced diffusion, some electrons will be able to diffuse up to about 150 μm from the cathode where the field is no longer negligible, and then easily be drifted away.

We suggest that the injection process is counterbalanced by the field recoiling, thus leading to a saturation of the current, *i.e.*, to stationary conditions, as indeed observed in Figure 9a. In summary, the analysis of the mechanisms going on under cathode irradiation is not straightforward: while transients tend to increase the slope of the electric field other effects, related to the charge injection and trap filling, superimpose.

As already mentioned, the current transient at 25 °C (Figure 9a) shows around 320 s an abrupt increase, which is definitely greater and steeper with respect to the smooth increases observed at the other temperatures. Such a feature, also observed for the 800 and 820 nm irradiations, is quite reproducible and it is probably still related to trap filling effects. After the seminal works of Lampert [29] and Rose [30] it is well known that current-voltage characteristic is profoundly affected by the crossing of a trap level by the Fermi level, that is, by the filling of a set of traps. Our results seem to indicate that such effects can also be observed in the time domain.

**Figure 11.**
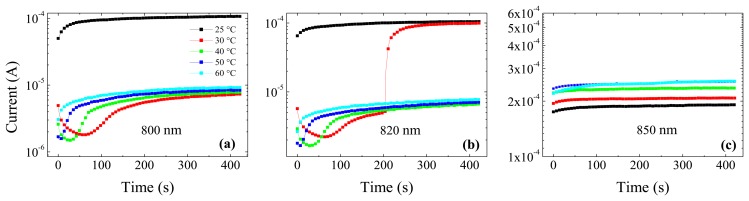
Current transients at different temperatures under cathode irradiation: (**a**) 800 nm; (**b**) 820 nm; (**c**) 850 nm. Applied voltage is 600 V.

Notably, the abrupt increase of the current follows a trend with temperature and wavelength: at 700 nm, as already observed in Figure 9a, the kink was evident only at 25 °C at about 320 s; at 800 nm (Figure 11a) it is also evident only at 25 °C, but already at the starting time of the transient; at 820 nm (Figure 11b) the sudden increase occurs not only in the transient at 25 °C but also at 30 °C, this time around 200 s from its beginning. Finally, at 850 nm (Figure 11c) it is as if the current is already in an *on-state* at the beginning of the transient, for all the tested temperatures. By skipping the surface defect region, the increase of the wavelength favours the carrier injection and the trap filling. On the other hand, the field slope increases with temperature, which makes the carrier injection from the cathode up to the edge of the field region less favourable at high temperatures.

It is clear that the large number of photo-generated carriers for irradiation at 850 nm and their dynamics have a deep impact on the field distribution and on the photocurrent. Next, we discuss some aspects related to such a strong perturbation on the CdTe Schottky detectors.

## 6. Photocurrent, Contacts and Deep Levels

The photocurrents measured under irradiations at 850 nm at the anode and the cathode deserve a few considerations in relation with the role of the contacts. When an electric field is applied between blocking contacts, as in the case of photodiodes, there is no gain effect and the maximum measured charge occurs for complete collection of the photo-generated carriers, or primary ones. This corresponds to the saturation of the collected charge commonly observed in Schottky and Ohmic CdTe detectors, when biased at sufficiently high voltages. This behaviour is described by the Hecht relation and is based on the Ramo's theorem. Continuous photo-generation correspondingly brings to a saturated photocurrent when transit time is shorter than trapping time. However, it is well known that in presence of Ohmic contacts, photoconductive gain occurs since the unbalancing of the electron/holes transport properties tends to leave a net positive charge inside the material. This forces the contact to inject negative charge (secondary carriers) thus producing a gain with respect to the photo-generated carriers. Unless other physical effects take place, e.g., carrier velocity saturation with high electric fields, carrier injection should result in a linear photocurrent *vs.* voltage response, surely not in a saturation (which in turns would be indicative of the absence of gain). Hence, the fully ‘Ohmic’ nature of contacts, like that of platinum electroless deposited on CdTe crystal used for detectors, becomes questionable since such detectors do not show gain.

On the other side, it is true that our data clearly show that electron injection from the Pt contact under high voltages occurs. In addition, the work function of platinum results in a very small band bending on semi-insulating CdTe, and this, in principle, is not the best condition to make an Ohmic contact. Hence, a kind of ‘limited’ Ohmic contact could exist, sufficiently Ohmic to inject a relatively low dc current but not enough to provide the secondary carriers, especially in pulsed experiments where currents are pretty high and therefore the contact could not be able to supply the requested carrier flux.

Another point concerns the high photocurrent values measured under the 850 nm irradiation which imply a high concentration of free carriers (both electrons and holes), inside the detector, about 100 μm beneath the irradiated surface. The presence of such a high concentration is also supported by the strong distortion of the electric field, which seems to indicate that free charges become comparable with the fixed space charges provided by deep levels. Under those circumstances, the quasi Fermi levels under optical irradiation move substantially towards their respective band edge and this in turns strongly influences the charge dynamics. In particular, the large amount of both kinds of carriers created along an extended region is subjected to recombination, either direct or through the deep levels, rather than to trapping.

Interestingly, it has been pointed out that the distinction between traps and recombination centres does not rely on their intrinsic properties only (as their carrier capture cross sections), but it can be roughly associated to the quasi-Fermi level energies. These features were clarified in the early analysis of photoconductivity in semiconductors [31], where the concept *of demarcation levels* was introduced: electron traps and recombination centres were defined as those above and below the electron demarcation level, respectively. On the basis of such consideration, we can infer that the high photocurrent values do not arise from the efficient extraction of electrons and holes, but from recombination between them.

The strong current saturation as a function of voltage, observed for anode irradiation at 850 nm, can now be interpreted in terms of an efficient recombination of the free carrier excess present in the region close to anode. Under anode irradiation, recombination only affects primary photo-generated carriers and the photocurrent saturation indicates that this mechanism is already efficient at low applied voltages, without injection contribution of secondary electrons from the cathode.

On the other hand, cathode irradiation at 850 nm produces a much higher photocurrent than anode irradiation, and the photocurrent does not saturate with voltage (see Figures 9 and 11). Irradiation at lower wavelengths has clearly evidenced features in I-Vs associated to electron injection and trap filling: in particular, the trap filling becomes apparent as a step-like process when voltage is increased. Moreover, the low field region close to the cathode contains a high concentration of free carriers which favours the electron injection. Hence, it appears that not only recombination between primary photo-generated carriers occurs, but the Ohmic contact is able, thanks to the high concentration of photo-generated carriers near the contact, to inject secondary electrons which fill the traps. Within this picture, the step-like features observed in I-Vs as well as the sudden increase in the current transients, should be attributed to secondary electrons injected by the contact, resulting in a gain.

In summary, for 850 nm irradiation on cathode side, recombination is still effective but also electron injection contributes to photocurrent, and this results in a weak but appreciable dependence on the voltage. The cathode, which under dark or anode irradiation behaves as a quasi ‘Ohmic’ contact, becomes now fully injecting, allowing gain to occur as in a standard photoconductor.

## 7. Conclusions

In this work we have shown that the Schottky CdTe X-ray detector is a complex system, where deep levels and contacts differently affect the electrical properties, especially under optical excitation. Our experimental approach provides significant insights since both electric field and current are rich in relevant and complementary information about the free and fixed charges inside the detector.

When voltage is applied in perturbation-free conditions (under dark), a negative space charge builds-up due to the hole-blocking anode contact, causing the field to fall linearly from such contact. With this regard, deep levels play a fundamental role: on one side they release carriers, causing a thermally activated shrinking of the electric field under the anode (*bias induced polarization*); on the other side, they provide, up to voltages corresponding to full depletion conditions, the main contribution to the flowing current, through thermal generation mechanism [27]. However, if voltage is increased such that the electric field is not negligible near the cathode, electron injection from the Ohmic contact becomes prevalent [27].

When anode or cathode are optically irradiated, the effect of the perturbation strongly depends not only on the incident wavelength, according to the different penetration depth, but also on the irradiation side. With respect to the wavelength, the following three cases can be identified:

(1) At short wavelengths (*λ* = 700, 800, 820 nm), when absorption is limited up to few microns, the defected region below the contacts subtracts photo-generated carries to transport, and the perturbation increases with wavelength. The small decrease of the electric field slope, observed under anode irradiation, is explained by the hole trapping. Hence, the space charge originating from deep levels ionization is still the main responsible for the perturbed electric field distribution, as highlighted from measurements at various temperatures. On the other hand, the relatively large increase of the slope observed under cathode irradiation is related to the injection of electrons from such contact. In the latter case, charge diffusion and trap filling effects become evident in the measured currents, with features similar to space charge limited currents that also appear during transient conditions. Only at high temperature (60 °C), the electric field under cathode irradiation is not perturbed, as it is still controlled by deep level carrier emission.
(2) The strongest perturbation is registered at wavelengths near the CdTe band edge (*λ* = 850 nm). Here the radiation is more penetrating, and much less affected by surface recombination; therefore, the large number of photo-generated electrons and holes plays the major role in the electric field distribution. In particular, a concavity is observed in the field profile, which has been attributed to the physical separation of the net charge, the positive holes prevailing on the cathode side. Results indicate that bulk carrier recombination becomes effective, and for cathode irradiation, a gain is possible, due to the injecting nature of cathode. Furthermore, reminding that our irradiation at 850 nm is equivalent to a high flux (1.7 × 10^10^ #/(mm^2^)) of 42 keV X-ray photons, we could infer that recombination plays a major role in the radiation induced polarization, by decreasing the effective lifetime.
  A relevant finding is that the origin of the radiation induced polarization is different from that of the bias induced polarization, *i.e.*, the space charge build-up.
(3) At longer wavelengths (*λ* = 900, 950 nm), corresponding to energies below the band gap, small but still appreciable perturbations have been observed. According to the low absorption coefficient, results are quite independent of the irradiation side and can be ascribed to transitions involving discrete levels in the energy gap. As a consequence of the direct space charge perturbation, a net increase of the electric field is observed. The role of defects in the transport mechanisms is strengthened by the thermal activation of the photocurrent. Future experiments will be performed under pulsed optical perturbing excitation. By looking at the charge/current single pulse response, we aim to quantify the lifetime perturbation in CdTe detectors.

## References

[1] Takahashi T., Mitani T., Kobayashi Y., Kouda M., Sato G., Watanabe S., Nakazawa K., Okada Y., Funaki M., Ohno R. (2002). High-resolution Schottky CdTe diode detector. IEEE Trans. Nucl. Sci..

[2] Toyama H., Higa A., Yamazato M., Maehama T., Ohno R., Toguchi M. (2006). Quantitative analysis of polarization phenomena in CdTe radiation detectors. Jpn. J. Appl. Phys..

[3] Cola A., Farella I. (2009). The polarization mechanism in CdTe Schottky detectors. Appl. Phys. Lett..

[4] Principato F., Gerardi G., Turturici A.A., Abbene L. (2012). Time-dependent current-voltage characteristics of Al/p-CdTe/Pt X-ray detectors. J. Appl. Phys..

[5] Vartsky D., Goldberg M., Eisen Y., Shamai Y., Dukhan R., Siffert P.M., Koebel J.M., Regal R., Gerber J. (1988). Radiation induced polarization in CdTe detectors. Nucl. Instrum. Method. Phys. Res. A.

[6] Du Y., LeBlanc J., Possin G.E., Yanoff B.D., Bogdanovich S. (2003). Temporal response of CZT detectors under intense irradiation. IEEE Trans. Nucl. Sci..

[7] Bale D.S., Szeles C. (2008). Nature of polarization in wide-bandgap semiconductor detectors under high-flux irradiation: Application to semi-insulating Cd(1-x)Zn(x)Te. Phys. Rev. B.

[8] Sato G., Fukuyama T., Watanabe S., Ikeda H., Ohta M., Ishikawa S., Shiraki H., Ohno R. (2011). Study of polarization phenomena in Schottky CdTe using infrared light illumination. Nucl. Instrum. Method. Phy. Res. A.

[9] Dědič V., Franc J., Sellin P.J., Grill R., Peruma V. (2012). Study on electric field in Au/CdZnTe/In detectors under high fluxes of X-ray and laser irradiation. J. Instrum..

[10] Washington A.L., Teague L.C., Duff M.C., Burger A., Groza M., Buliga V. (2012). Wavelength dependence on the space charge collection in CdZnTe detectors. J. Appl. Phys..

[11] Siffert P.M., Berger J., Scharager C., Cornet A., Stuck R., Bell R.O., Serreze H.B., Wald F.V. (1976). Polarization in cadmium telluride nuclear radiation detectors. IEEE Trans. Nucl. Sci..

[12] Prokesh M., Bale D.S., Szeles C. (2010). Fast high-flux Response of CdZnTe X-ray detectors by optical manipulation of deep level occupations. IEEE Trans. Nucl. Sci..

[13] De Antonis P., Morton E.J., Podd F.J.W. (1996). Infra-red microscopy of Cd(Zn)Te radiation detectors revealing their internal electric field structure under bias. IEEE Trans. Nucl. Sci..

[14] Zumbiehl A., Hage-Ali M., Fourgeres P., Koebel J.M., Regal R., Siffert P. (1999). Electric field distribution in CdTe and Cd_1-x_Zn_x_Te nuclear detectors. J. Cryst. Growth.

[15] Khusainov K., Antonova Y.A., Lysenlko V.V., Makhkamov R.K., Morozov V.Z., Ilves A.G., Arlt R.D. (2001). Energy resolution of large-area CdTe p-i-n detectors with charge loss corrections. Nucl. Instrum. Method. Phys. Res. A.

[16] Cola A., Farella I., Auricchio N., Caroli E. (2006). Investigation of the electric field distribution in X-ray detectors by Pockels effect. J. Opt. A Pure Appl. Opt..

[17] Sellin P.J., Prekas G., Franc J., Grill R. (2010). Electric field distributions in CdZnTe due to reduced temperature and X-ray irradiation. Appl. Phys. Lett..

[18] Franc J., Dědič V., Sellin P.J., Grill R., Veeramani P. (2011). Radiation induced control of the electric field in Au/CdTe/In structures. Appl. Phys. Lett..

[19] Cola A., Farella I., Mancini A.M., Donati A. (2007). Electric field properties of CdTe nuclear detectors. IEEE Trans. Nucl. Sci..

[20] Uxa S., Belas E., Grill R., Praus P., James R.B. (2012). Determination of electric-field profile in CdTe and CdZnTe detectors using transeint-current technique. IEEE Trans. Nucl. Sci..

[21] Suzuki K., Sawada T., Imai K., Seto S. (2012). Time-of-flight measurements on Schottky CdTe detectors. IEEE Trans. Nucl. Sci..

[22] Namba S. (1961). Electro-optical effect of zincblende. J. Opt. Soc. Am..

[23] Cola A., Farella I., Anni M., Martucci M.C. (2012). Charge transients by variable wavelength optical pulses in CdTe nuclear detectors. IEEE Trans. Nucl. Sci..

[24] Cuzin M. (1987). Some new developments in the field of high atomic number materials. Nucl. Instrum. Method. Phys. Res. A.

[25] De Montmorillon L.A., Delaye P., Launay J.C., Roosen G. (1995). Comparative study of CdTe and GaAs performances from 1 μm to 1.55 μm. Opt. Mater..

[26] Ramo S. (1939). Currents induced by electron motion. Proc. I.R.E..

[27] Cola A., Farella I. (2013). Electric fields and dominant carrier transport mechanisms in CdTe Schottky detectors. Appl. Phys. Lett..

[28] Zanichelli M., Pavesi M., Marchini L., Zappettini A. (2012). Studies on charge collection and transport properties on semi-insulating materials in the presence of a non-uniform electric field. Solid State Commun..

[29] Lampert L.A. (1956). Simplified theory of space-charge-limited currents in an insulator with traps. Phys. Rev..

[30] Rose A. (1955). Space-charge-limited currents in solids. Phys. Rev..

[31] Rose A. (1963). Concepts in Photoconductivity and Allied Problems.

